# Quantum dots get a bright upgrade

**DOI:** 10.1038/s41377-024-01593-0

**Published:** 2024-09-23

**Authors:** Fei Ding

**Affiliations:** Institut für Festkörperphysik, Leibniz Universität Hannover, Appelstraße 2, 30167 Hannover, Germany

**Keywords:** Quantum dots, Quantum optics

## Abstract

Developing a bright, deterministic source of entangled photon pairs has been an outstanding scientific and technological challenge. Semiconductor quantum dots are a promising candidate for this task. A new device combining a circular Bragg resonator and a piezoelectric actuator achieves high brightness and entanglement fidelity simultaneously, overcoming previous limitations. This breakthrough enhances quantum dot applications in entanglement-based quantum communication protocols.

Scalable sources of entangled photons are the keystone for realizing photonic quantum networks and enabling entanglement-based quantum communication protocols. Currently, most sources are based on the spontaneous parametric down-conversion (SPDC) process, which are fundamentally limited by their probabilistic photon emission, hindering their scalability and widespread adoption.

Semiconductor quantum dots have emerged as promising candidates to overcome these limitations (see Fig. 1). Quantum dots can generate polarization-entangled photon pairs on-demand^1,2^, with high photon flux, indistinguishability, and entanglement fidelity^3^. However, the key challenge has been that optimizing different figures of merit, like brightness (extraction efficiency or photon pair rate per excitation pulse) and entanglement fidelity, often requires different technological solutions that are difficult to be integrated into single devices.

**Fig. 1:**
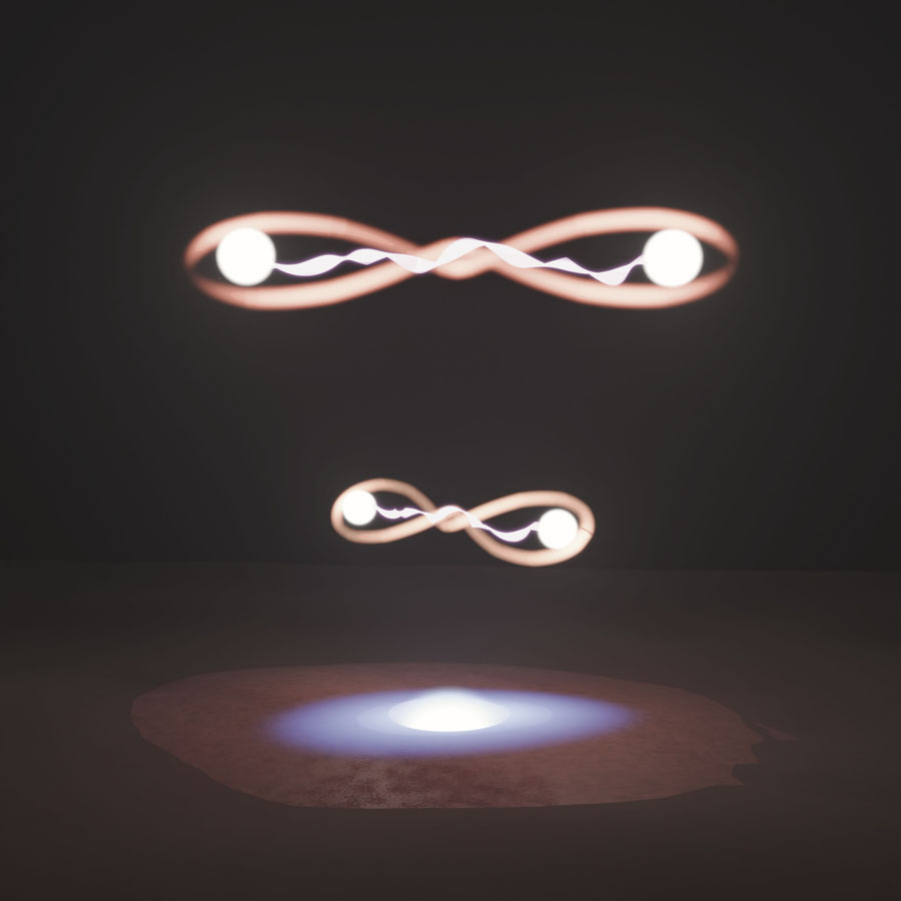
Artistic perception of entangled photon pairs generated from a single quantum dot

Previous efforts illustrate these trade-offs. Dousse et al.^4^ increased the photon pair generation rate to 0.12 per excitation pulse by coupling quantum dots to micropillar photonic molecules^4^. This was further pushed to 0.65 by Liu et al.^3^ by using quantum dots embedded in circular ring gratings^3^. However, the lack of tunability in these devices limited the achievable entanglement fidelity. Work from Huber et al.^5^ used strain-tuning to generate highly entangled photon pairs from GaAs quantum dots, with fidelities up to 0.98, but at the cost of modest brightness^5^.

Attempts at combining brightness enhancement and entanglement optimization proved difficult. Now, a team of researchers from Sapienza University of Rome, Johannes Kepler University Linz, and the University of Würzburg have successfully developed a powerful device that overcomes this long-standing conundrum. Their approach, described in a recent paper in eLight^6^, combines two cutting-edge technologies - a circular Bragg resonator structure^7^ that enhances the brightness of the quantum dot emission, and an integrated piezoelectric actuator^8^ that allows precise tuning of strain fields^9^ to maximize the entanglement.

This is a beautiful demonstration that both high brightness and high entanglement can be achieved simultaneously from a quantum dot based entangled photon source. This device can emit entangled photon pairs with fidelities up to 0.96, very close to the maximum value, while still maintaining a photon pair rate of 0.69.

The key was optically positioning and then embedding the quantum dot inside a specially engineered circular Bragg grating resonator. The fabricated photonic structure accelerates the dot’s emission via Purcell enhancement and funnels a large fraction of emitted photons into a collimated beam, building on earlier works like that of Davanco et al.^7^ and Liu et al.^3^. Simultaneously, finely controlled strain fields from the piezoelectric actuator allowed canceling out the energetic fine structure splitting that normally scrambles the photon entanglement, similar to the approach used by Plumhof et al.^10^.

This breakthrough, based on the hybrid resonator-actuator approach, directly tackles the long-standing challenge of simultaneously optimizing brightness and entanglement for quantum dot sources, and may finally lead to a source that checks all the boxes, i.e., high brightness, high fidelity entanglement, and even high single photon indistinguishability. This is crucial for enabling the exploitation of semiconductor quantum dots in entanglement-based quantum network protocols.

Although the device has reached a very high level of complexity and functionality, there is still great potential for improvement. The strain tuning range can be significantly improved by thinning down the GaAs nanomembrane that contains the quantum dots. The single photon indistinguishability can be boosted further by stabilizing the charge environment and reducing spectral wandering with a charge tunable diode^11^, and moreover, by controlling the timing correlations between the entangled photon pairs^12^. Non-zero multiphoton emission probabilities and residual components in the reconstructed density matrix may also be improved in future devices. To fully harness the potential of this semiconductor platform, one should also consider including electrical injection (in order to replace the bulky laser excitation setups)^13,14^, and/or integrating full electrical control to tune the wavelength of entangled photons as demonstrated recently by Chen et al.^15^. Among the next steps, realizing entanglement swapping^16,17^ with two remote sources would be a key experiment to prove the scalability of quantum dots based sources.

From early work on micropillar photonic molecules to the latest electrical wavelength-tuning capabilities, the multi-decade quest to unleash the potential of semiconductor quantum dots as entangled photon sources has seen steady progress. The focus of the community is gradually shifting from physics curiosity to useful quantum technology out of the lab^18–21^. We believe that quantum dots will truly become an enabling technology for realizing quantum networks and all their revolutionary applications.

## References

[1] Benson, O. et al. Regulated and entangled photons from a single quantum dot. *Phys. Rev. Lett.* **84**, 2513–2516 (2000). 11018923 10.1103/PhysRevLett.84.2513

[2] Stevenson, R. M. et al. A semiconductor source of triggered entangled photon pairs. *Nature* **439**, 179–182 (2006). 16407947 10.1038/nature04446

[3] Liu, J. et al. A solid-state source of strongly entangled photon pairs with high brightness and indistinguishability. *Nat. Nanotechnol.* **14**, 586–593 (2019). 31011221 10.1038/s41565-019-0435-9 PMC10941235

[4] Dousse, A. et al. Ultrabright source of entangled photon pairs. *Nature* **466**, 217–220 (2010). 20613838 10.1038/nature09148

[5] Huber, D. et al. Strain-tunable GaAs quantum dot: a nearly dephasing-free source of entangled photon pairs on demand. *Phys. Rev. Lett.* **121**, 033902 (2018). 30085806 10.1103/PhysRevLett.121.033902

[6] Rota, M. B. et al. A source of entangled photons based on a cavity-enhanced and strain-tuned GaAs quantum dot. *eLight* **4**, 13 (2024). 39070906 10.1186/s43593-024-00072-8 PMC11269457

[7] Davanço, M. et al. A circular dielectric grating for vertical extraction of single quantum dot emission. *Appl. Phys. Lett.* **99**, 041102 (2011).

[8] Ding, F. et al. Tuning the exciton binding energies in single self-assembled InGaAs/GaAs quantum dots by piezoelectric-induced biaxial stress. *Phys. Rev. Lett.* **104**, 067405 (2010). 20366855 10.1103/PhysRevLett.104.067405

[9] Trotta, R. et al. Energy-tunable sources of entangled photons: a viable concept for solid-state-based quantum relays. *Phys. Rev. Lett.* **114**, 150502 (2015). 25933298 10.1103/PhysRevLett.114.150502

[10] Plumhof, J. D. et al. Strain-induced anticrossing of bright exciton levels in single self-assembled GaAs/Al_*x*_Ga_1-*x*_As and In_*x*_Ga_1-*x*_As/GaAs quantum dots. *Phys. Rev. B* **83**, 121302(R) (2011).

[11] Zhai, L. et al. Low-noise GaAs quantum dots for quantum photonics. *Nat. Commun.* **11**, 4745 (2020). 32958795 10.1038/s41467-020-18625-z PMC7506537

[12] Schöll, E. et al. Crux of using the cascaded emission of a three-level quantum ladder system to generate indistinguishable photons. *Phys. Rev. Lett.* **125**, 233605 (2020). 33337175 10.1103/PhysRevLett.125.233605

[13] Zhang, J. X. et al. High yield and ultrafast sources of electrically triggered entangled-photon pairs based on strain-tunable quantum dots. *Nat. Commun.* **6**, 10067 (2015). 26621073 10.1038/ncomms10067 PMC4686767

[14] Yuan, Z. L. et al. Electrically driven single-photon source. *Science* **295**, 102–105 (2001). 11743163 10.1126/science.1066790

[15] Chen, C. et al. Wavelength-tunable high-fidelity entangled photon sources enabled by dual Stark effects. *Nat. Commun.* **15**, 5792 (2024). 38987247 10.1038/s41467-024-50062-0 PMC11237044

[16] Zopf, M. et al. Entanglement swapping with semiconductor-generated photons violates Bell’s inequality. *Phys. Rev. Lett.* **123**, 160502 (2019). 31702338 10.1103/PhysRevLett.123.160502

[17] Basset, F. B. et al. Entanglement swapping with photons generated on demand by a quantum dot. *Phys. Rev. Lett.* **123**, 160501 (2019). 31702339 10.1103/PhysRevLett.123.160501

[18] Lu, C. Y. & Pan, J. W. Quantum-dot single-photon sources for the quantum internet. *Nat. Nanotechnol.* **16**, 1294–1296 (2021). 34887534 10.1038/s41565-021-01033-9

[19] Basso Basset, F. et al. Quantum key distribution with entangled photons generated on demand by a quantum dot. *Sci. Adv.* **7**, eabe6379 (2021). 33741595 10.1126/sciadv.abe6379 PMC7978422

[20] Zahidy, M. et al. Quantum key distribution using deterministic single-photon sources over a field-installed fibre link. *npj Quantum Inf.* **10**, 2 (2024).

[21] Yang, J. Z. et al. High-rate intercity quantum key distribution with a semiconductor single-photon source. *Light Sci. Appl.* **13**, 150 (2024). 38956020 10.1038/s41377-024-01488-0 PMC11219984

